# Real-World Efficacy of HLX02-Based Neoadjuvant Therapy in HER2-Positive Breast Cancer: Clinical Insights and Future Directions

**DOI:** 10.1155/tbj/1653319

**Published:** 2025-07-10

**Authors:** Zhengzhi Zhu, Jing Wang, Shikai Hong, Hong Gao, Jianjun Liu, Kuojun Ren, Shuhan Wang, Shengying Wang, Guoping Sun

**Affiliations:** ^1^Department of Oncology, The First Affiliated Hospital of Anhui Medical University, Hefei, Anhui, China; ^2^Department of Breast Center, Anhui Provincial Cancer Hospital, West District of The First Affiliated Hospital of University of Science and Technology of China, Hefei, China

**Keywords:** anti-HER2 therapy, breast cancer, CDK4/6 inhibitor, HER2-positive, HLX02, HR-positive

## Abstract

**Background:** The efficacy of HLX02, a trastuzumab biosimilar, in combination with chemotherapy for treating metastatic breast cancer (BC) has been established as equivalent to the reference Herceptin. This study aimed to assess the treatment response of HLX02-based neoadjuvant therapy in HER2-positive BC, with a focus on HR-positive versus HR-negative subgroups. Additionally, we investigated the potential role of a CDK4/6 inhibitor in combination with anti-HER2 therapy.

**Methods:** This retrospective study included HER2-positive BC patients who received HLX02-based neoadjuvant therapy followed by curative surgery at Anhui Provincial Cancer Hospital between March 2021 and August 2023. Pathological complete response (pCR) rates were analyzed, and subgroup analyses evaluated predictors of pCR. In vitro experiments using BT-474 and MCF-7 cell lines assessed the effects of combining CDK4/6 inhibitors with anti-HER2 therapy on cell viability and apoptosis.

**Results:** The study included 67 patients with a median age of 53 years. The overall pCR rate was 53.73%, with higher pCR rates observed in HR-negative patients compared to HR-positive patients (63.89% vs. 41.94%). Dual HER2 blockade with HLX02 and pertuzumab was associated with a numerically improved pCR rate (62.16%). ER expression significantly increased post-treatment, potentially indicating treatment resistance mechanisms. In vitro, the combination of CDK4/6 inhibitors with anti-HER2 therapy significantly reduced cell viability and promoted apoptosis in HR-positive, HER2-positive cell lines.

**Conclusion:** HLX02 demonstrates real-world efficacy as part of neoadjuvant therapy for HER2-positive BC, especially in HR-negative patients. The lower pCR rate in HR-positive patients highlights the need for additional strategies. Combining CDK4/6 inhibitors with anti-HER2 therapy presents a promising approach for HR-positive HER2-positive patients, warranting further clinical validation.

## 1. Introduction

Breast cancer (BC) is the most common malignancy among women, with approximately 2.3 million new cases reported worldwide in 2022, significantly impacting the physical and mental well-being of patients and representing one of the most pressing global health challenges [1]. BC is highly heterogeneous, with distinct molecular subtypes exhibiting varied tumor behaviors and prognoses. Approximately 20% of cases involve HER2 overexpression (HER2-positive), known for its aggressive nature and higher risk of recurrence [2, 3]. Over the past 30 years, targeted HER2 therapies have revolutionized the prognosis for these patients, particularly those diagnosed at an early stage, enabling long-term survival and even potential cure. However, it has become increasingly apparent that the efficacy of HER2-targeted treatment is not uniformly ideal for all HER2-positive patients [4]. Notably, about half of the HER2-positive population is also hormone receptor (HR) positive, defined by the presence of estrogen receptor (ER) and/or progesterone receptor (PgR) [5, 6]. These dual HER2-positive/HR-positive tumors pose unique treatment challenges due to dual pathway involvement, contributing to complex disease behavior and suboptimal responsiveness to HER2-targeted therapies [4].

The NOAH trial demonstrated that adding the anti-HER2 antibody trastuzumab to standard neoadjuvant therapy significantly improved the pathological complete response (pCR) rate and increased the 3-year event-free survival (EFS) rate to over 70% for HER2-positive patients [7]. This pivotal trial marked the beginning of the era of anti-HER2 neoadjuvant therapy. Today, HER2-targeted neoadjuvant therapy has become the standard treatment for patients with early-stage HER2-positive BC, irrespective of HR status. The NeoSphere trial further validated the efficacy of anti-HER2 neoadjuvant therapy and introduced a dual-targeted regimen combining trastuzumab, pertuzumab, and chemotherapy, which achieved a pCR rate of 45.8% (95% CI 36.1–55.7) among all HER2-positive patients [8]. Notably, HER2-positive/HR-negative patients showed a numerically higher pCR rate of 63.2% (95% CI 49.3–75.6), suggesting that the cross-talk between hyperactive HR and HER2 pathways significantly influences tumor biology and patient outcomes. Additionally, achieving pCR has been associated with markedly improved survival compared to non-pCR outcomes [9]. These observations underscore the ongoing debate regarding the optimal treatment strategy for HR-positive HER2-positive BC, highlighting the need for tailored approaches based on BC subtypes. Although endocrine therapy (ET) is the mainstay for HR-positive patients, studies have demonstrated the bidirectional cross-talk between the HER2 and ER pathways, with HER2 overexpression related to endocrine resistance [10]. The efficacy of CDK4/6 inhibitors has been well-established in HR-positive, HER2-negative BC [11]; however, their potential benefit when combined with HER2-targeted therapies in HER2-positive patients remains unclear.

At the same time, the economic burden associated with novel therapies has limited the widespread use of trastuzumab, especially in low- and middle-income countries, leaving many patients to face poor physical and mental outcomes due to inadequate treatment access [12]. HLX02 (marketed as 汉曲优 in China, HERCESSI in the US, and Zercepac in Europe), a trastuzumab biosimilar to Herceptin, is a monoclonal antibody targeting HER2 receptors with significant potential to curb tumor proliferation [13]. Approved by the European Medicines Agency (EMA), the China National Medical Products Administration (NMPA), and the United States Food and Drug Administration (FDA), HLX02 has broadened access to HER2-targeted therapy in most parts of the world. Although its bioequivalence to trastuzumab has been well-documented, more real-world studies are necessary to fully understand the clinical efficacy of HLX02 in routine practice.

To address these challenges, we conducted a retrospective clinical analysis combined with in vitro cellular studies to explore the real-world clinical efficacy of neoadjuvant combination therapy based on HLX02 in patients with HER2-positive early-stage BC, with a particular focus on the HR-positive subgroup. Additionally, we evaluated the antiproliferative activity of trastuzumab combined with palbociclib, a CDK4/6 inhibitor, using HER2-positive, HR-positive cell models. This research aims to provide a robust evidence base for the clinical application of HLX02 and investigate the potential of combination therapy in preclinical models, offering insights that could inform and refine treatment strategies for HER2-positive BC, especially for patients with HR-positive disease.

## 2. Materials and Methods

### 2.1. Participants

A retrospective study was conducted at Anhui Provincial Cancer Hospital. Female patients diagnosed with HER2-positive BC between March 2021 and August 2023 were identified from the electronic medical record database. Eligible patients were aged 18 years or older, had histologically confirmed HER2-positive BC, received HLX02 or/and pertuzumab in combination with chemotherapy for neoadjuvant therapy, and subsequently underwent radical mastectomy. There were no other restrictions on the specific anti-HER2 neoadjuvant regimen used. Patients with other malignant tumors or insufficient clinical data were excluded from the analysis. Demographic and clinicopathological characteristics of the patients were collected. This study was approved by the Ethics Committee of the Anhui Provincial Cancer Hospital (No. 2021–19), with a waiver of written informed consent from patients. This study was conducted in accordance with the Declaration of Helsinki.

### 2.2. Outcomes

The primary endpoint of the retrospective analysis was the pCR, defined as no invasive cancer in the breast and lymph nodes, with only in situ residuals allowed in the breast tissue postsurgery. Tumor pathology was also assessed using the Miller–Payne (MP) grading system [14] and the residual cancer burden (RCB) index. The tumor response during neoadjuvant therapy was evaluated according to the Response Evaluation Criteria in Solid Tumors version 1.1 (RECIST v1.1), which categorizes responses into CR, partial response (PR), stable disease (SD), and progressive disease (PD). The objective response rate (ORR) is calculated as the proportion of patients achieving either CR or PR. Additionally, changes in the expression of ER, PgR, and Ki67-index in patients with residual cancer were analyzed.

### 2.3. Cell Lines and Intervention

MCF-7 and BT-474 cell lines were obtained from the Shanghai Institute of Biochemistry and Cell Biology (SIBCB). MCF-7 cells were maintained in DMEM (Gibco, 11,965,092) supported with 10% fetal bovine serum (FBS; Gibco, A5669701) and 1% penicillin-streptomycin (Gibco, 15,070,063). BT-474 cells were maintained in RPMI 1640 medium (Gibco, 11,875,093) with 10% FBS and 1% penicillin-streptomycin. Both cell lines were cultured at 37°C in a humidified atmosphere containing 5% CO_2_.

For the intervention, cells were seeded into plates containing the appropriate complete medium and allowed to adhere overnight. The following treatments were then applied for 48 h: trastuzumab (an anti-HER2 mAb; Henlius, HERCESSI/Zercepac) at a concentration of 10 μg/mL alone, palbociclib (a CDK4/6 inhibitor; Selleck, S1116) at a concentration of 0.5 μg/mL alone, and a combination of both 10 μg/mL trastuzumab and 0.5 μg/mL palbociclib. Each treatment was performed in triplicate and the entire experiment was repeated three times.

### 2.4. Cell Viability and Apoptosis Assays

Cell viability was evaluated using the Cell Counting Kit-8 (CCK-8; Beyotime, C0038). Cells were plated at a density of 2000 cells per 100 μL in 96-well plates and incubated overnight to allow for adhesion. Post-adhesion, cells underwent intervention with monoagent and combination treatments, followed by a 48-h incubation at 37°C. After incubation, 10 μL of CCK-8 solution was added to each well, and the mixture was incubated for an additional 2 h. Absorbance was measured at 450 nm to determine optical density (OD) values.

For apoptosis assessment, the TdT-mediated dUTP Nick-End Labeling (TUNEL; Beyotime, C1091) assay was used. Cells were seeded at 5 × 10^4^ cells per 500 μL of culture medium in each well of a 24-well plate. After 48 h of treatment, cells were washed with phosphate-buffered saline (PBS), fixed with 4% paraformaldehyde for 30 min, and permeabilized with 0.2% Triton X-100. The TUNEL reaction was then conducted according to the manufacturer's protocol. Apoptotic cells were quantified using ImageJ software.

### 2.5. Statistical Methods

Statistical analyses were performed using R software (version 4.3.2). Categorical variables were presented as numbers and percentages (%), while numerical variables were described as mean ± standard deviation (SD) or median (range). Univariable and multivariable logistical regression analyses were conducted to identify predictors for pCR, with results expressed as odds ratio (OR) and 95% confidence interval (CI). Variables with a *p* value ≤ 0.10 in univariable analysis were included in the multivariable logistic regression model. Nonparametric statistical tests were employed to evaluate the changes in ER, PgR, and Ki67-index following neoadjuvant treatment. Differences in OD values between groups were tested using the *t*-test. Unless otherwise specified, a significance level of 0.05 was used for all statistical tests.

## 3. Results

### 3.1. Baseline Characteristics

Through a comprehensive review of electronic medical records, we identified 312 early-stage BC patients treated at our hospital between March 2021 and August 2023. Of these, 67 HER2-positive female patients who received HLX02-based neoadjuvant therapy followed by curative surgery were included in the final analysis (Supporting Figure 1). The median age of the patients was 53 years (range, 21–69), with the majority being postmenopausal (*n* = 44, 65.67%) (Table 1). Most tumors exhibited invasive growth patterns (*n* = 62, 92.54%), with more than half occurring in the left breast (*n* = 44, 65.67%) and predominantly in the upper outer quadrant (*n* = 40, 59.70%). At the initiation of neoadjuvant therapy, the majority of patients (*n* = 55, 82.09%) had stage II disease, and 31 patients (46.27%) were HR-positive. Over half of the cohort (*n* = 37, 55.22%) received dual HER2-targeted therapy with HLX02 and pertuzumab. Specifically, 29 patients (43.28%) were treated with the TCbHP regimen (docetaxel, carboplatin, HLX02, pertuzumab), while 8 patients (11.94%) received the THP regimen (docetaxel, HLX02, pertuzumab).

### 3.2. Efficacy and Subgroup Analysis of HLX02-Based Neoadjuvant Therapy

**Figure 1:**
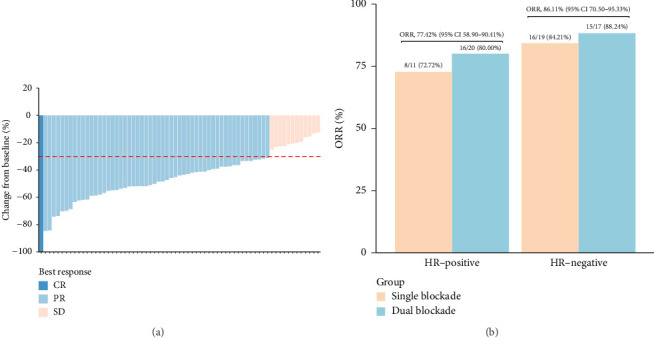
Tumor response during HLX02-based neoadjuvant therapy: (a) Best change from baseline in target lesion size for HER2-positive patients following HLX02-based neoadjuvant therapy. (b) Tumor response stratified by hormone receptor (HR) status and treatment with dual HER2 blockade (trastuzumab and pertuzumab) according to RECIST 1.1 criteria.

During neoadjuvant therapy, all patients experienced tumor shrinkage. PR was observed in 54 patients, and 1 patient achieved a CR, resulting in an ORR of 82.09% (95% CI 70.80%–90.39%) (Figure 1(a), Table 2). Among the 31 HR-positive patients, 20 (64.52%) received dual-targeted therapy with trastuzumab and pertuzumab, and 24 achieved PR, yielding an ORR of 77.42% (95% CI 58.90%–90.41%). In contrast, among the 36 HR-negative patients, 17 (47.22%) received dual-targeted therapy, and the ORR was 86.11% (95% CI 70.50%–95.33%) (Figure 1(b)). This suggests that, in real-world clinical practice, the treatment regimens for HER2-positive/HR-positive patients tend to be more intensive but may not overcome the inherent resistance associated with HR positivity in anti-HER2 therapy.

**Figure 2:**
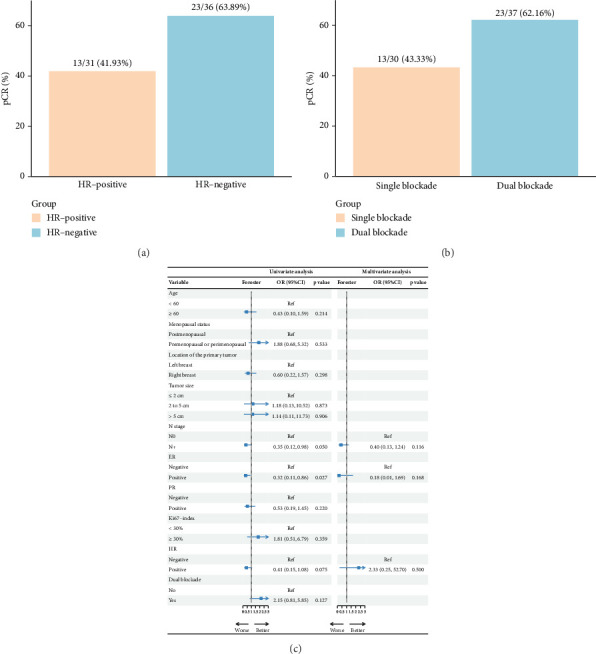
Pathological response and subgroup analysis following neoadjuvant therapy: (a) Pathological complete response (pCR) stratified by hormone receptor (HR) status. (b) pCR stratified by treatment with dual HER2 blockade (trastuzumab and pertuzumab). (c) Subgroup analysis of pCR outcomes.

pCR was achieved in 36 patients (53.73%), with HR-negative patients showing a numerically higher pCR rate compared to HR-positive patients (63.89% vs. 41.94%) (Figure 2(a), Table 2). Among the 37 patients who received dual-targeted therapy, 23 (62.16%) achieved pCR, compared to 13/30 (43.33%) who received trastuzumab-only regimens (Figure 2(b)). Additionally, 36 patients (53.73%) were classified as G5 according to the MP grading system, and 39 (58.21%) had an RCB score of 0 (Table 2). The discrepancy between MP grading and RCB assessment was attributed to three patients with missing or unassessed MP data. Furthermore, subgroup analysis revealed that ER positivity was associated with a lower rate of pCR (OR = 0.32; 95% CI 0.11–0.86; *p*=0.027) (Figure 2(c)). No other significant positive findings were observed in the subgroup analyses.

### 3.3. Changes in Tumor Biomarker Expression Pre- and Postneoadjuvant Therapy

For the 31 patients who did not achieve pCR, we analyzed the changes in tumor expression of ER, PgR, and the Ki67 index before and after neoadjuvant therapy. Following HER2-targeted neoadjuvant treatment, a significant increase in ER expression was observed (from 60.00% to 76.82%; *p*=0.038), while the Ki67 index showed a significant decrease (from 40.00% to 19.76%; *p*=0.002) (Table 3). However, changes in PgR expression were not statistically significant (*p*=0.173) (Table 3). Additionally, we noted that four non-pCR patients who were HR-negative before treatment exhibited HR-positive status after neoadjuvant therapy. This change could be attributed to the spatial heterogeneity of intratumoral molecular expression or might represent a potential mechanism of resistance to HER2-targeted treatment.

### 3.4. In Vitro Efficacy of Combined CDK4/6 Inhibition and Anti-HER2 Therapy

**Figure 3:**
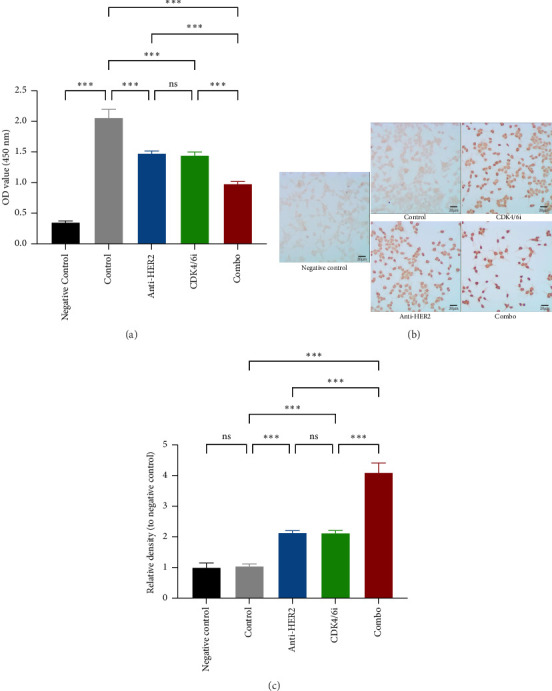
Effects of anti-HER2, CDK4/6 inhibitor, and combination treatments on BT-474 cell viability and apoptosis: (a) The CCK-8 assay results show changes in BT-474 cell viability under different treatments. (b) Representative images of TUNEL staining in BT-474 cells for each treatment group. (c) A bar graph comparing the differences in TUNEL staining across treatment groups. In the CCK-8 assay, the negative control consisted of wells without cells, incubated with CCK-8 reagent, while in the TUNEL assay, the negative control included cells not incubated with the TUNEL reaction mixture. The control group was treated with a solvent for 48 hours. The anti-HER2 group was treated with 10 μg/mL trastuzumab for 48 h, the CDK4/6i group was treated with 0.5 μg/mL palbociclib for 48 h, and the combo group was treated with 10 μg/mL trastuzumab and 0.5 μg/mL palbociclib for 48 h. The results indicate that the combination treatment significantly reduced cell viability and increased apoptosis compared to either treatment alone. ^∗∗∗^, *p* < 0.001; ns, not significant.

To investigate potential enhancements in treatment efficacy for HR-positive HER2-positive BC, we conducted in vitro studies assessing the impact of CDK4/6 inhibitors combined with HER2-targeted therapy on tumor cell behavior. The BT-474 cell line was employed to evaluate the effects on cell viability and apoptosis. The CCK-8 assay revealed that combining a CDK4/6 inhibitor with anti-HER2 therapy significantly reduced cell viability compared to anti-HER2 monotherapy (*p* < 0.001) and CDK4/6 inhibitor monotherapy (*p* < 0.001) (Figure 3(a)). Consistently, the TUNEL assay demonstrated a notable increase in apoptosis with the combination treatment compared to either anti-HER2 therapy alone (*p* < 0.001) or CDK4/6 inhibition alone (*p* < 0.001) (Figures 3(b) and 3(c)). To corroborate these findings, parallel analyses were performed using the MCF-7 cell line. The results aligned with observations from BT-474 cells, supporting the potential efficacy of combined therapy. Due to the variable reports on HER2 expression in MCF-7 cells—some studies indicating positivity and others showing negativity [15, 16]—data from these experiments are included in Supporting Figure 2 for comprehensive context.

## 4. Discussion

This retrospective real-world study reaffirmed the efficacy of HLX02 as a trastuzumab biosimilar in the neoadjuvant treatment setting for HER2-positive BC. Our findings indicated that more than half of the patients achieved pCR after treatment (overall pCR rate: 53.73%). Although consistent with prior studies, this pCR rate was numerically lower than those reported in other single-center retrospective analyses, which documented pCR rates of 70.8% and 68.2% for patients receiving the TCbHP and THP regimens, respectively [17, 18]. The lower pCR rate in our study could be attributed to the higher proportion of HR-positive patients, known to have lower pCR rates following neoadjuvant therapy [19], as well as the inclusion of various HLX02-based regimens, potentially impacting outcome uniformity. It should be noted that, like the prior analyses, our study was single-center and retrospective, potentially limiting the robustness and generalizability of the findings. However, the consistent evidence across studies supports the clinical use of HLX02 as an effective alternative to reference trastuzumab. Notably, our study's diverse treatment regimens reflect real-world clinical scenarios, further supporting its practical relevance. Additionally, patients receiving dual HER2 blockade with trastuzumab and pertuzumab achieved a 62.16% pCR rate, aligning with the results from the NeoSphere trial, which demonstrated the enhanced efficacy of dual HER2-targeted therapy combined with chemotherapy in improving outcomes for HER2-positive patients [8]. A meta-analysis of 11 randomized controlled trials (RCTs) also confirmed that dual HER2 blockade is associated with higher pCR rates in nonmetastatic HER2-positive BC (OR 2.88, 95% CI: 2.02–4.10) [20]. However, our subgroup analysis did not show a significant association between dual blockade and pCR, likely due to confounding factors inherent in real-world research. Larger studies are needed to validate the efficacy of dual HER2 blockade in routine clinical practice.

Our study further highlighted that HR status influences the effectiveness of anti-HER2 therapy, with HR-positive patients showing lower pCR rates. While current evidence suggests the combination of ET treatment for HR-positive patients, ET is often associated with notable adverse effects and decreased quality of life, especially in premenopausal women. As a result, during the short window of neoadjuvant therapy, patients typically receive anti-HER2 treatment alone to minimize delays or cancellations of surgery due to ET intolerance, with ET introduced during adjuvant therapy. Moreover, existing research indicates that primary resistance to ET is common in HR-positive/HER2-positive BC. Despite these findings, the crosstalk between HER2 and HR pathways remains poorly understood [4]. Previous investigators have identified an inverse correlation between HER2 overexpression and ER expression at the protein level [21]. The 2016 subanalysis of the HERA trial also suggested that *ERBB2* (the gene encoding HER2) amplification levels are generally lower in HR-positive, HER2-positive BC patients [22]. Mechanistically, research indicates that ER can phosphorylate the mitogen-activated protein kinase (MAPK) and members of the growth factor receptor family, including fibroblast growth factor receptor (FGFR) and epithelial growth factor receptor (EGFR), while HER2 can activate ER through tyrosine kinase domain phosphorylation [23, 24]. This cross-talk can drive aggressive tumor behavior and contribute to resistance and treatment escape [25]. In our study, we observed that four patients who did not achieve pCR exhibited a shift from HR-negative to varying levels of ER and PgR expression after HER2-targeted therapy. Data from HER2-positive cell lines indicated that ER or its downstream signaling targets increased after treatment with lapatinib and trastuzumab [26]. Giuliano et al. [27] found that 18% of ER-negative patients were converted to ER-positive after 2 weeks of neoadjuvant lapatinib treatment. This phenomenon was also observed in clinical studies, where HR-negative metastatic patients exhibited a sudden upregulation of ER following chronic exposure to trastuzumab [28], and similar findings during anti-HER2 treatment have been reported in another real-world study [29]. This phenomenon could be attributed to spatial heterogeneity in intratumoral molecular expression, leading to discrepancies in HR status before and after treatment. Alternatively, it is possible that changes in HR status are related to resistance mechanisms associated with HER2-targeted therapy, potentially representing a mechanism of resistance.

The development of antibody-drug conjugates (ADCs) has introduced new therapeutic approaches. The ADAPT trial, for example, showed that neoadjuvant treatment with T-DM1, with or without ET, resulted in a 41% pCR rate in HR-positive, HER2-positive patients [30]. However, despite the potential of novel treatments, their development and clinical validation are lengthy, leaving many patients at risk of recurrence, progression, or death before benefiting from these advancements. Immediate priorities in clinical practice include identifying accessible and effective combination therapies. To address this need, we investigated the addition of the CDK4/6 inhibitor palbociclib to anti-HER2 therapy in HR-positive, HER2-positive cell lines. Our in vitro results showed that dual inhibition of HER2 and CDK4/6 significantly reduced cell viability and induced apoptosis. CDK4/6 inhibitors, known to control cell cycle progression, have shown robust antitumor activity in HR-positive, HER2-negative BC, significantly prolonging progression-free survival (PFS) and overall survival (OS) in advanced disease [31, 32]. However, their efficacy in HER2-positive patients, particularly in the neoadjuvant setting, remains uncertain. Supporting our findings, Nikolai et al. demonstrated that CDK4/6 inhibitors could inhibit proliferation in HER2-overexpressing BC cells by modulating E2F1 and its target genes, which are involved in HER2-driven DNA metabolism [33]. Mechanistically, CDK4/6 phosphorylates the retinoblastoma (RB) protein, a tumor suppressor protein, which induces limited activation of transcriptional regulator E2F, and then stimulates the expression of downstream proteins required for passing into the S phase via the G1 checkpoint [34, 35]. The MonarcHER trial, which included 273 patients with HER2-positive/HR-positive advanced BC, found that the combination of fulvestrant, abemaciclib, and trastuzumab improved patient outcomes compared to trastuzumab plus chemotherapy [36]. The SOLTI-1303 PATRICIA study also observed promising efficacy with anti-HER2 therapy combined with CDK4/6 inhibition in this patient population [37]. Nevertheless, due to the retrospective nature of our study, we were unable to assess the tolerability and potential antitumor activity of this combination in the neoadjuvant setting.

Previous evidence has established a correlation between pCR after neoadjuvant therapy and improved survival outcomes [9]. However, prospective research has suggested that in HR-positive/HER2-positive patients, lower pCR rates following anti-HER2 treatment may not adversely impact long-term survival [38, 39], likely due to the benefit derived from extended adjuvant hormone therapy lasting at least 1 year. Our study did not include long-term follow-up to assess patient outcomes beyond the neoadjuvant setting, due to challenges in patient tracking, and resource constraints. This limitation means we could not determine the long-term prognostic impact of treatment. It is noteworthy that real-world patient management often faces significant challenges, including poor adherence to long-term ET, which could negatively influence survival.

Undoubtedly, our study has several limitations. First, the real-world, retrospective design may introduce inherent biases. We included only patients who underwent curative surgery following neoadjuvant therapy, resulting in the exclusion of patients who experienced disease progression during treatment. The relatively small sample size further underscores the need for additional evidence to corroborate our findings. Second, our analysis was limited to patients treated with HLX02, as the reference trastuzumab was not readily accessible before HLX02 received approval. This limited treatment accessibility may have impacted the generalizability of our results. Although variations in chemotherapy regimens may introduce potential bias, subgroup analyses were not feasible in this analysis due to insufficient sample sizes within specific treatment subgroups, warranting larger studies. Additionally, due to the generally favorable prognosis of early-stage BC, long-term follow-up poses significant challenges, preventing us from assessing long-term survival benefits. Finally, although we observed promising in vitro efficacy of combined HER2 and CDK4/6 inhibition, we were unable to explore the clinical effectiveness of this strategy in the neoadjuvant setting due to the absence of prospective studies. Despite these limitations, our research holds significant clinical value. It provides real-world evidence supporting the use of HLX02, a globally approved trastuzumab biosimilar, thereby enhancing the accessibility of anti-HER2 therapies for more BC patients. Furthermore, this study may inspire future investigations into novel neoadjuvant strategies for HER2-positive/HR-positive BC.

## 5. Conclusion

In summary, our study demonstrated that most HER2-positive BC patients, particularly those with HR-negative status, benefit significantly from anti-HER2 neoadjuvant therapy (HLX02 and/or pertuzumab in combination with chemotherapy). The observed lower pCR rate among HR-positive patients and the significant upregulation of ER expression underscore the need for innovative combination therapies to enhance treatment efficacy for this subset. The combination of CDK4/6 inhibitors with anti-HER2 therapy showed potential as a promising approach for HR-positive, HER2-positive patients by significantly reducing tumor cell viability and promoting apoptosis in preclinical models. These findings suggest that tailoring therapeutic strategies based on specific molecular profiles is crucial for optimizing treatment outcomes. However, further high-quality clinical studies are required to thoroughly investigate the efficacy and safety of such combination regimens in clinical practice.

## Figures and Tables

**Table 1:** Baseline and clinical characteristics of patients.

Characteristics	Total (*N* = 67)
Age (years)	
< 60, *n* (%)	56 (83.58)
≥ 60, *n* (%)	11 (16.42)
Median (range)	53 (21–69)
Tumor growth patterns, *n* (%)	
Invasive	62 (92.54)
Other	5 (7.46)
T stage, *n* (%)	
T1	5 (7.46)
T2	51 (76.12)
T3	11 (16.42)
N stage, *n* (%)	
N0	45 (67.16)
N1	16 (23.88)
N2	3 (4.48)
N3	3 (4.48)
M stage, *n* (%)	
M0	66 (98.51)
M1^a^	1 (1.49)
Clinical stage, *n* (%)	
I	4 (5.97)
II	55 (82.09)
III	7 (10.45)
IV^a^	1 (1.49)
Menopausal status, *n* (%)	
Postmenopausal	44 (65.67)
Premenopausal or perimenopausal	23 (34.33)
Location of the primary tumor, *n* (%)	
Left breast	44 (65.67)
Right breast	23 (34.33)
Location of the target lesion, *n* (%)	
Upper outer quadrant	40 (59.70)
Lower outer quadrant	11 (16.42)
Upper inner quadrant	9 (13.43)
Lower inner quadrant	4 (5.97)
Central region	2 (2.99)
Other	1 (1.49)
Tumor size, *n* (%)	
≤ 2 cm	4 (5.97)
> 2 cm, ≤ 5 cm	48 (71.64)
> 5 cm	15 (22.39)
Comorbidities, *n* (%)	7 (10.45)
Hypertension	6 (8.96)
Coronary heart disease	1 (1.49)
Ki67-index, *n* (%)	
< 30%	12 (17.91)
≥ 30%	55 (82.09)
HR-positive, *n* (%)	31 (46.27)
ER-positive	27 (40.30)
PgR-positive	25 (37.31)
Therapy regimen^b^, *n* (%)	
TCbHP	29 (43.28)
TCbH	22 (32.84)
TCbHPy	6 (8.96)
THP	8 (11.94)
TCH	2 (2.99)
Dual blockade^c^, *n* (%)	
Yes	37 (55.22)
No	30 (44.78)

*Note:* PgR, progesterone receptor.

Abbreviations: ER, estrogen receptor; HR, hormone-receptor.

^a^One patient had oligometastatic disease at baseline. Upon evaluation by the treating physician, the primary tumors were potentially resectable. The patient received neoadjuvant therapy and subsequently underwent radical mastectomy.

^b^TCbHP (docetaxel, carboplatin, HLX02, pertuzumab), TCbH (docetaxel, carboplatin, HLX02), TCbHPy (docetaxel, carboplatin, HLX02, pyrotinib), THP (docetaxel, HLX02, pertuzumab), TCH (docetaxel, cyclophosphamide, HLX02).

^c^The dual blockade refers to those treated with trastuzumab and pertuzumab.

**Table 2:** Tumor response to HLX02-based neoadjuvant therapy.

	Total (*N* = 67)	Dual blockade (*n* = 37)	Single blockade (*n* = 30)
Best overall response			
CR	1 (1.49)	1 (2.70)	0 (0.00)
PR	54 (80.60)	30 (81.08)	24 (80.00)
SD	12 (17.91)	6 (16.22)	6 (20.00)
ORR (95% CI)	82.09 (70.80–90.39)	83.78 (67.99, 93.81)	80.00 (64.13–92.29)
Pathological response			
pCR	36 (53.73)	23 (62.16)	13 (43.33)
MP classification			
G1	1 (1.49)	1 (2.70)	0 (0.00)
G2	7 (10.45)	5 (13.51)	2 (6.67)
G3	3 (4.48)	1 (2.70)	2 (6.67)
G4	14 (20.90)	6 (16.22)	8 (26.67)
G5	36 (53.73)	23 (62.16)	13 (43.33)
Missing	6 (8.96)	1 (2.70)	5 (16.67)
RCB classification			
0	39 (58.21)	23 (62.16)	16 (53.33)
I	5 (7.46)	2 (5.41)	3 (10.00)
II	17 (25.37)	8 (21.62)	9 (30.00)
III	5 (7.46)	3 (8.11)	2 (6.67)
Missing	1 (1.49)	1 (2.70)	0 (0.00)

**Table 3:** Changes of ER, PgR, and Ki67 following HLX02-based neoadjuvant therapy.

Expression	Before treatment	After treatment	*p* value
ER	60.00% ± 36.27%	76.82% ± 24.87%	**0.038**
PgR	30.00% ± 31.87%	19.53% ± 20.34%	0.173
Ki67-index	40.00% ± 14.58%	19.76% ± 14.10%	**0.002**

*Note:* Data are displayed as mean ± standard deviation (SD). Bold values indicate statistical significance was determined at *p* < 0.05.

## Data Availability

The data that support the findings of this study are available on request from the corresponding author. The data are not publicly available due to privacy or ethical restrictions.

## Supporting Information

Supporting Figure 1. Flowchart of patient screening.

Supporting Figure 2. Effects of Anti-HER2, CDK4/6 Inhibitor, and Combination Treatments on MCF-7 Cell Viability and Apoptosis.
