# Supplementary material for: Real-World Efficacy of HLX02-Based Neoadjuvant Therapy in HER2-Positive Breast Cancer: Clinical Insights and Future Directions
Source: Breast J. 2025 Jul 10;2025:1653319. doi: 10.1155/tbj/1653319 (PMC12271716; doi:10.1155/tbj/1653319)
Supplement: Supporting Information — Additional supporting information can be found online in the Supporting Information section. [file 1653319.f1.docx]

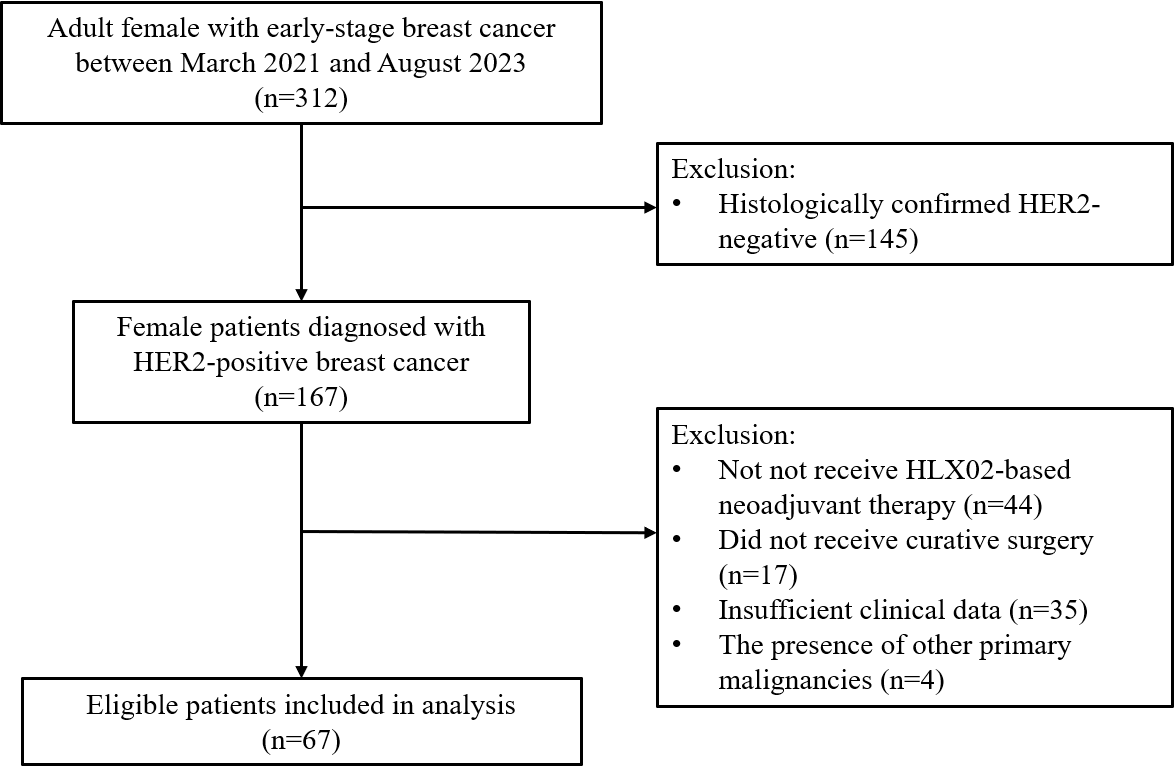


**Supplementary Figure 1. Flowchart of patient screening**


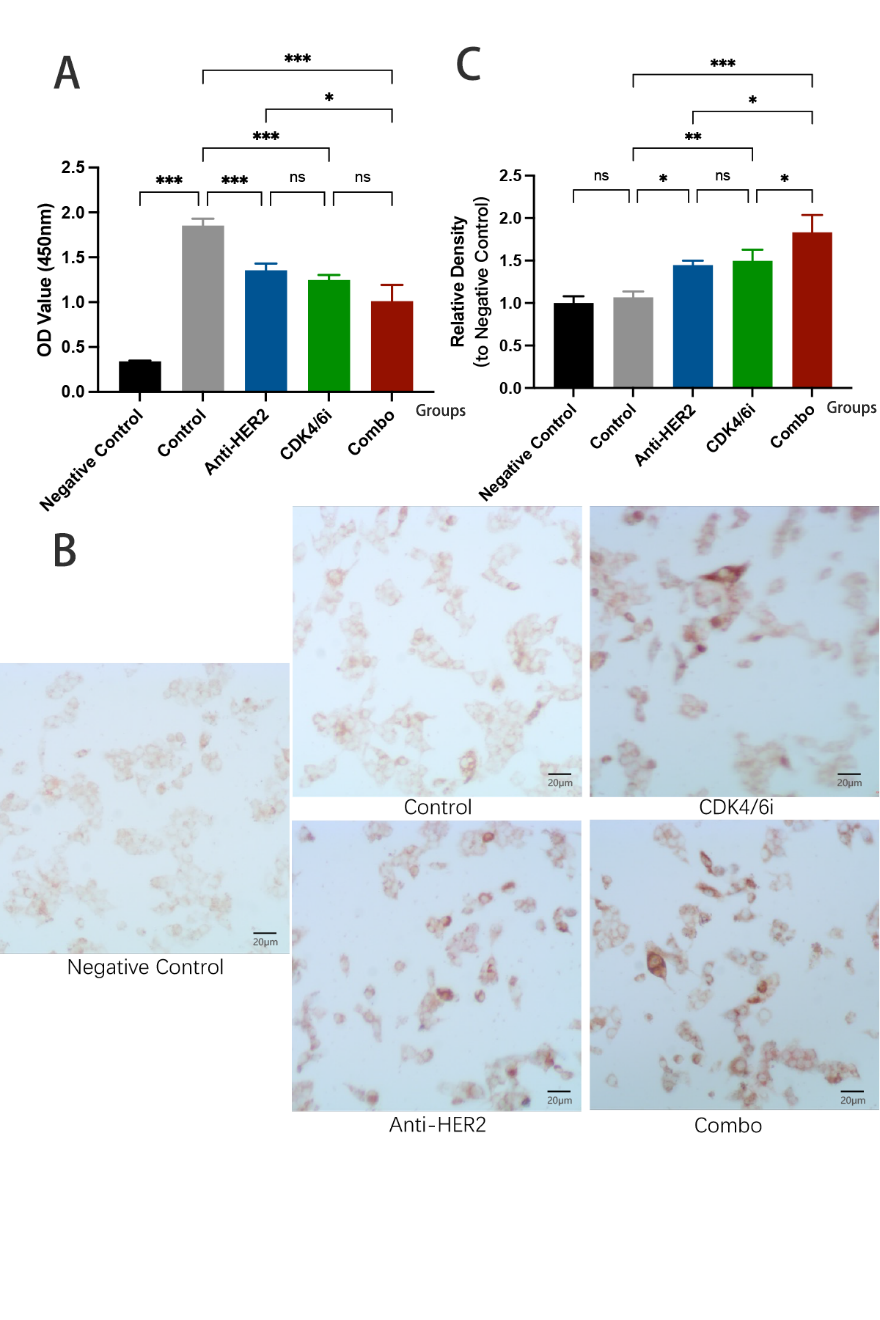


**Supplementary Figure 2. Effects of Anti-HER2, CDK4/6 Inhibitor, and Combination Treatments on MCF-7 Cell Viability and Apoptosis**

A. The CCK-8 assay results show changes in MCF-7 cell viability under different treatments. B. Representative images of TUNEL staining in MCF-7 cells for each treatment group. C. A bar graph comparing the differences in TUNEL staining across treatment groups.

In the CCK-8 assay, the negative control consisted of wells without cells, incubated with CCK-8 reagent, while in the TUNEL assay, the negative control included cells not incubated with the TUNEL reaction mixture. The control group was treated with a solvent for 48 hours. The anti-HER2 group was treated with 10 μg/ml trastuzumab for 48 hours, the CDK4/6i group was treated with 0.5 μg/ml palbociclib for 48 hours, and the combo group was treated with 10 μg/ml trastuzumab and 0.5 μg/ml palbociclib for 48 hours. The results indicate that the combination treatment significantly reduced cell viability and increased apoptosis compared to either treatment alone. *, *P*<0.05; **, *P*<0.01; ***, *P*<0.001; ns, not significant.
